# Neovaginal perforation following sexual intercourse in a transsexual patient

**DOI:** 10.1186/1756-0500-7-797

**Published:** 2014-11-15

**Authors:** Hasan Deliktas, Onder Ozcan, Nesat Cullu, Omer Erdogan

**Affiliations:** Department of Urology, School of Medicine, Mugla Sitki Kocman University, Mugla, 48000 Turkey; Department of Surgery, School of Medicine, Mugla Sitki Kocman University, Mugla, Turkey; Department of Radiology, School of Medicine, Mugla Sitki Kocman University, Mugla, Turkey

**Keywords:** Neovaginal perforation, Sexual intercourse, Transsexual

## Abstract

**Background:**

Neovaginal perforation can develop following sexual intercourse in patients that have undergone male to female gender reassignment surgery. In such cases urinary tract symptoms may mimic acute cystitis and acute pyelonephritis.

**Case presentation:**

A 33-year old white transsexual patient presented to the emergency department with dysuria, hematuria, difficulty urinating, widespread groin pain, bilateral side pain, clear vaginal discharge, abdominal pain, and nausea 2-3 h after sexual intercourse. Abdominal tomography showed fluid around the vaginal cuff and air throughout the abdomen. Vaginography showed contrast leaking to the abdomen from the vaginal cuff. The patient was considered as vaginal perforation and admitted to clinic.

**Conclusion:**

Vaginal perforation should be considered in transsexual patients that develop urinary system symptoms following sexual intercourse. Such cases were treated medically without the need surgery.

## Background

In male to female gender reassignment surgery penile and scrotal skin graft, or the rectosigmoid intestinal segment can be used to create a neovagina [1, 2]. Creation of a neovagina using penile and scrotal skin graft is associated with more scar tissue and insufficient vaginal cuff length [3, 4]; therefore, the likelihood of vaginal perforation following sexual intercourse is elevated in such cases. Herein we present the radiological findings and treatment in a 33-year old gender reassignment surgery patient with vaginal perforation.

## Case presentation

A 33-year old white transsexual patient presented to the emergency department with dysuria, hematuria, difficulty urinating, widespread groin pain, bilateral side pain, clear vaginal discharge, abdominal pain, and nausea 2-3 h after sexual intercourse. The patient had undergone male to female gender reassignment surgery 7 years earlier, in which penile and scrotal skin graft was used to create a neovagina. Physical examination showed that there was sensitivity in the suprapubic region, costovertebral angle sensitivity, and defense and rebound.

**Figure 1:**
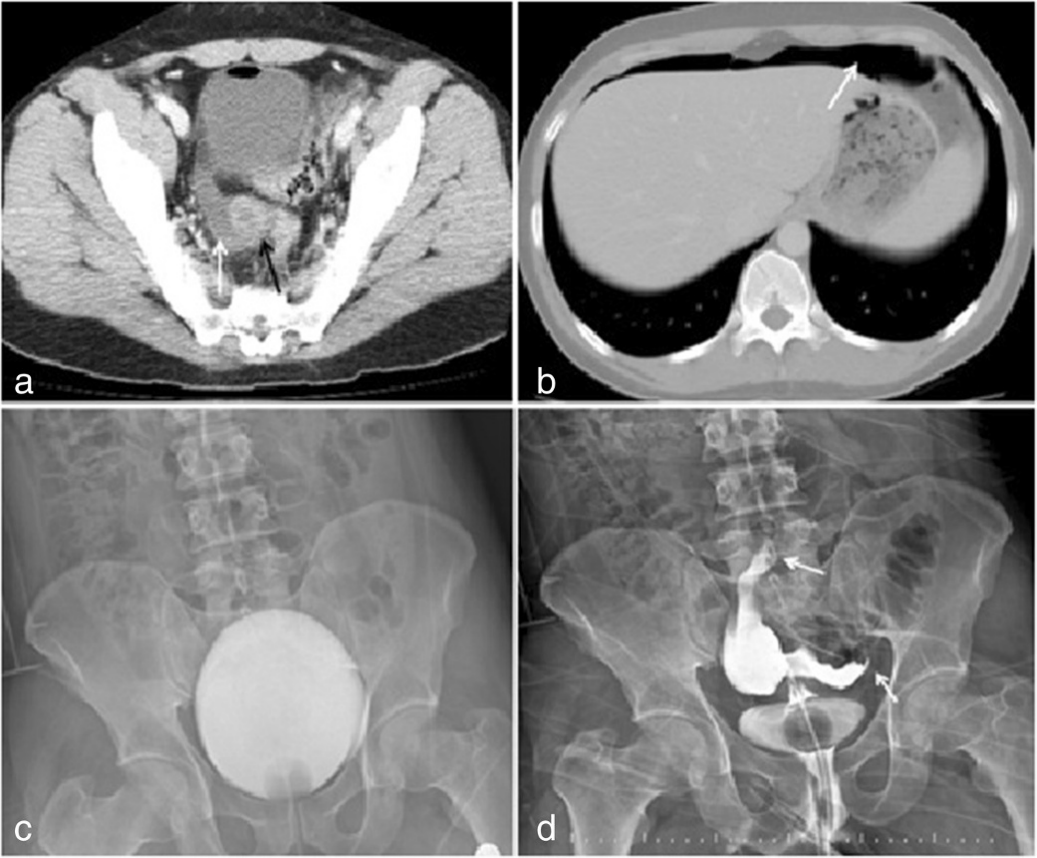
**Axial pelvic tomography image; free fluid (white arrow) around the vaginal cuff (black arrow) (a).** Axial abdominal tomography image; Free air (white arrow) in the subdiaphragmatic region **(b)**. Cystography image; Contrast extravasation was not observed **(c)**. Vaginography image; Contrast leaking (white arrow) into the abdomen from the vaginal cuff **(d)**.

Laboratory findings included red blood cells 120 u/L in the full urine test and leukocytes 19 × 10^9^ cells/L in the full blood count; other laboratory findings were normal. Abdominal ultrasonography showed fluid around the vaginal cuff and abdominal tomography showed fluid around the vaginal cuff and air throughout the abdomen (Figure 1a and b). As vaginal perforation was considered, cystography was performed to search for extravasation originating from the bladder, but extravasation was not observed (Figure 1c). Examination by the gynecology department showed clear discharge localized in the neovagina. A vaginal contrast examination was performed. Vaginography showed contrast leaking to the abdomen from the vaginal cuff (Figure 1d).

The patient’s abdominal pain, examination findings, and laboratory findings were evaluated by a specialist general surgeon. The patient was admitted to clinic. As there was no pathology other than vaginal perforation based on abdominal ultrasonography and abdominal tomography, and as the neovagina appeared normal upon vaginal examination, it was thought that with close monitoring the patient would recover and a combination of ceftriaxone 1000 mg b.i.d. and metronidazole 500 mg t.i.d. antibiotherapy was started and continued for 7 d. The patient’s abdominal pain became less severe and the fluid discharge from the neovagina stopped. On d 4 of the treatment the leukocyte count returned to normal, and on d 7 of the treatment the patient was discharged with oral antibiotic therapy. At the 1-month follow-up examination the patient was problem-free. At 6 weeks post treatment the patient recommenced sexual intercourse and at the 6-month follow-up had no complaints.

## Discussion

Penile and scrotal skin graft is the most common method for creating a neovagina, as it is associated with the lowest complication rate and is the easiest to perform; however, there are some disadvantages to the method, such as scar formation, an insufficient vaginal cavity, a narrow vaginal, intravaginal hair, the need for continuous dilatation, and the need for lubrication for sexual intercourse [3, 4]. The literature includes 2 case reports of neovaginal perforation. Amirian *et al*. [5] reported a case of neovaginal perforation after washing with a douche pump and Giovanni Liguari *et al*. [6] reported spontaneous perforation in the neovagina after stenosis. To the best of our knowledge the present case report is the first to describe neovaginal perforation following sexual intercourse. In addition, in the 2 earlier cases the sigmoid colon segment was used for the creation of the neovagina. In the presented case scrotal and penile skin graft was used, and to the best of our knowledge this is the first report of neovaginal perforation in a neovagina formed from scrotal and penile skin graft.

Franco *et al*. reported that in transsexual individuals in which penile and scrotal skin graft was used neovaginal length was 8-10 cm, which is longer than the natural vagina [7]; however, even with a vagina of 8-10 cm created using penile and scrotal skin graft the expansion and extension potential of the neovagina in those cases were lower than those of a normal vagina and those created using the sigmoid segment. As such, the likelihood of vaginal perforation during sexual intercourse is higher in such cases. In transsexual surgery sacrospinous ligament fixation of the neovagina is performed in order to prevent prolapse during the postoperative period [8]. By inhibiting the mobilization capability of the neovagina such fixation facilitates perforation during sexual intercourse.

In the presented case there was an indication for laparotomy because of the acute abdominal findings; however, as the patient had undergone surgery 7 years earlier it was thought that performing laparotomy again could have resulted in additional complications. Moreover, the neovaginal perforation case reported by Amirian *et al*. was followed up conservatively and the patient recovered without any surgical intervention [5].

As seen in previous studies on transsexual females in which penile skin was used to create the neovagina, the flora of the neovagina is formed of aerobic and anaerobic microorganisms in the mixed structure found in skin and intestinal flora [9, 10]. In the presented case administration of antibiotherapy that was effective on both aerobic and anaerobic microorganisms, and close monitoring resulted in complete recovery without the need for surgery.

## Conclusion

In transsexual patients that present with urinary system symptoms following sexual intercourse vaginal perforation should always be a consideration. Minimal perforations in such patients can be treated medically, without surgery.

## Consent

Written informed consent was optained from the patient for publication of this case report and any accompanying images. A copy of the written consent is available for review by the Editor of this journal.
